# Control of Persistent *Salmonella* Infection Relies on Constant Thymic Output Despite Increased Peripheral Antigen-Specific T Cell Immunity

**DOI:** 10.3390/pathogens9080605

**Published:** 2020-07-25

**Authors:** J. Alan Goggins, Jonathan R Kurtz, James B. McLachlan

**Affiliations:** Department of Microbiology and Immunology, Tulane University School of Medicine, New Orleans, LA 70112, USA; jgoggins@tulane.edu (J.A.G.); jkurtz1@tulane.edu (J.R.K.)

**Keywords:** *Salmonella*, CD4^+^ helper T cells, thymus, IL-17, persistent infection, recent thymic emigrants

## Abstract

Recent thymic emigrants are the youngest subset of peripheral T cells and their involvement in combating persistent bacterial infections has not been explored. Here, we hypothesized that CD4^+^ recent thymic emigrants are essential immune mediators during persistent *Salmonella* infection. To test this, we thymectomized adult mice either prior to, or during, persistent *Salmonella* infection. We found that thymic output is crucial in the formation of protective immune responses during the early formation of a *Salmonella* infection but is dispensable once persistent *Salmonella* infection is established. Further, we show that thymectomized mice demonstrate increased infection-associated mortality and bacterial burdens. Unexpectedly, numbers of *Salmonella*-specific CD4^+^ T cells were significantly increased in thymectomized mice compared to sham control mice. Lastly, we found that T cells from thymectomized mice may be impaired in producing the effector cytokine IL-17 at early time points of infection, compared to thymically intact mice. Together, these results imply a unique role for thymic output in the formation of immune responses against a persistent, enteric pathogen.

## 1. Introduction

The thymus serves as the anatomical site where new cytotoxic (CD8^+^) and helper (CD4^+^) T cells initially express the T cell receptor (TCR). After these newly formed T cells have been trained to recognize self-major histocompatibility complex (MHC) (positive selection) and self-antigen-reactive T cells have been eliminated (negative selection), they are released into the periphery as a specialized population of T cells known as recent thymic emigrants (RTEs) which take 1–3 weeks to become the fully mature, but naïve T cells that are normally thought to constitute the bulk of peripheral antigen-reactive T cells [1,2]. It is also believed that thymic output and the production of RTEs early in life helps contribute new TCR diversity to the peripheral lymphocyte pool [3], theoretically increasing the number of antigens that can be recognized during an immune response. It is known that thymic function is greatly influenced by peripheral events, particularly events that cause significant immunological stress. Viral, bacterial and parasitic infections have all been shown to induce thymic involution [4,5], in addition to environmental stressors, sex hormones and starvation [6,7]. Much of the impact of infection on the thymus has been attributed to increased systemic levels of glucocorticoids (GC) and pro-inflammatory cytokines, which have a negative impact on thymopoiesis and induce acute thymic atrophy. Many of these outcomes are temporary and are generally resolved upon cessation of the immunological stress [6].

Whether thymic output serves a continuous protective role against infection after the peripheral lymphocyte pool has been established is not well understood. While there are studies exploring how infection impacts the function of the thymus itself, many studies are performed in mice thymectomized at birth [8,9,10,11,12]. Little is known about how thymic function impacts infection outcomes in adult animals. Few studies have explored the role of thymic output during bacterial infections and even these are conflicting. One study found that thymectomized, wild-type adult mice exhibited an impairment of bacterial clearance upon a secondary challenge against *Listeria monocytogenes*, compared to mice with an intact thymus [13]. Another study using a murine infection model of *Mycobacterium lepraemurium* demonstrated that adult thymectomy increased bacterial clearance showing that the presence of the thymus appeared to worsen infection outcomes [14]. These types of conflicting findings leave the field unclear in terms of the role of the thymus during bacterial infection.

In humans, infection with *Salmonella enterica* serovar Typhi (*S.* Typhi) causes the disease typhoid fever, which is endemic in many developing countries [15]. Approximately 10% of people recovering from untreated typhoid fever may excrete *S.* Typhi in their stool for at least 3 months [16]. Between 1% and 5% of all typhoid patients become chronic carriers, defined by excretion of *S.* Typhi in urine or stool for more than one year [17]. Chronic carrier rates are observed to be higher in women, patients 50 years or older and patients with co-morbidities such as schistosomiasis, cholelithiasis, carcinoma of the gall bladder and other gastrointestinal malignancies [18]. Most chronic carriers remain asymptomatic and estimates state that as many as 25% of all chronic carriers may have had no history of typhoid fever [16]. *S.* Typhi is known to be a strict human pathogen. However, other serovars are more broadly host adapted and capable of causing disease in both animals and humans. *Salmonella enterica* serovar Typhimurium for example causes a typhoid-like systemic and persistent disease in certain mouse strains but results in acute gastroenteritis in humans [19]. This persistent state in mice reliably mimics chronic Typhoid fever in humans and thus can be used as a model to understand the interplay between the host immune system and the bacteria throughout the course of this persistent carriage state [20,21,22].

Thymic output may especially be important during *Salmonella* infection, where CD4^+^ T cells have been shown to be essential for resistance against both acute and persistent infection [22,23,24,25]. While it is known that *Salmonella* infection induces thymic atrophy [12], to our knowledge, no one has specifically investigated the impact of the thymus itself on *Salmonella* infection outcomes, particularly during persistent infection. Furthermore, it is not known whether the peripheral T cell response to persistent *Salmonella* infection is affected, either positively or negatively, by the loss of thymic output. It is also unclear whether *Salmonella*-specific peripheral T cells, when RTEs are absent, change their phenotype in response to infection.

In the study reported here, we sought to answer the question of whether continuous thymic output was important for protecting mice against persistent *Salmonella* infection and whether the *Salmonella*-specific T cells were altered in response to infection when the thymus was absent. We found that, indeed, thymectomized adult mice could not control persistent *Salmonella* infection despite a surprising, and significant, increase in peripheral *Salmonella*-specific CD4 T cells. Further, these T cells appeared to be less capable of producing the cytokine IL-17, which may play a role in controlling bacterial infection. These findings point to an as yet unknown function for RTEs in protecting mice against a persistent *Salmonella* infection. 

## 2. Results

### 2.1. Mice Lacking a Thymus at the Initiation of Infection Succumb to Oral Salmonella Infection

It is known that control of both acute and persistent *Salmonella* infection relies on the presence of CD4 T cells [22,23,24,25]. What is not known is whether existing peripheral CD4 T cells are sufficient to control infection or whether a continuous supply of new T cells is important for controlling bacterial infection. We initially set out to answer the question of whether thymic output plays a role in controlling persistent *Salmonella* infection. To do this we performed thymectomies on adult 129Sv x C57Bl/6 first-generation crossed mice (hereafter F1 mice) which carry a functional copy of the natural resistance associated macrophage protein 1 (NRAMP-1) gene (known as *Slc11a1*). Functional NRAMP-1 is essential for controlling intracellular *Salmonella* growth making common mouse strains used for *Salmonella* infection (e.g., C57BL/6), harboring a G169D substitution in NRAMP-1, susceptible to lethal *Salmonella* infection [26,27]. Notably, F1 mice are resistant to *Salmonella* infection and can be used to mimic the persistent *Salmonella* infectious carriage state seen in human typhoid fever [17,18,22]. We thymectomized F1 mice at 6 weeks of age when thymic output peaks followed by reproductive maturity and subsequently thymic involution [2]. We also allowed for a three-week post surgical recovery period to permit the resolution of surgical inflammation, as well as the full maturation of any remaining RTEs in the peripheral compartment [1,2].

**Figure 1:**
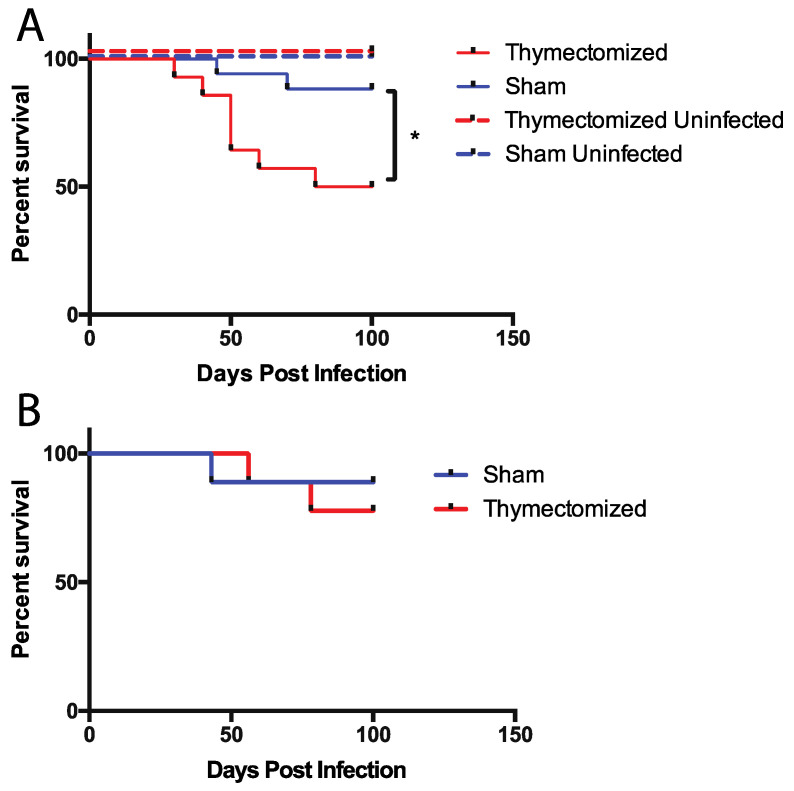
Thymectomy prior to *Salmonella* infection leads to increased mortality in adult mice. (**A**) Adult F1 mice underwent thymectomy (n = 14) or sham surgery (n = 17). Three weeks later, mice were orally infected with WT *Salmonella* (5 × 10^7^ CFU). As a control for mortality associated with surgical treatment alone, mice were subjected to full thymectomy (n = 5) or sham surgical control (n = 5) treatment. Survival was measured up to 100 days post infection. (**B**) Adult F1 mice were infected as in A. Thirty days after infection, mice were subjected to either full thymectomy (n = 9) or sham surgical control treatment (n = 9). Survival was measured up to 100 days post surgery and was statistically analyzed using the Gehan–Brelow–Wilcoxon test for all groups. * = *p* < 0.02. Representative of two independent experiments combined.

Thymectomized and sham surgical control (to mimic the inflammation caused by surgery) F1 mice were then orally infected with 5 × 10^7^ colony forming units (CFU) of virulent, wild-type *Salmonella* strain SL1344. We then tracked *Salmonella*-associated mortality for 100 days post infection. We observed significantly increased mortality over time in infected, thymectomized mice compared to infected sham surgical control mice that had an intact thymus (Figure 1A). It was possible that thymectomy alone induced the observed mortality independent of infection. To test this, we performed thymectomies or sham surgery as before and monitored mortality over time without infecting the mice with *Salmonella* (Figure 1A). In this case, we did not observe any mortality associated with thymectomy in the absence of infection indicting that the combination of a lack of thymic output in the face of infection led to the observed mortality. We next hypothesized that inhibiting thymic output while infection was ongoing would also cause mice to succumb to infection. To examine this, we thymectomized mice 30 days after the initiation of *Salmonella* infection; however, surprisingly, we did not observe the same differences in *Salmonella*-associated mortality as we did when mice were thymectomized prior to infection (Figure 1B), indicating that thymic function and output may be important during the initiation of *Salmonella* infection. However, this output may be dispensable once infection has been established. 

### 2.2. Preinfection Thymectomy Results in Significantly Increased Salmonella Burdens Systemically Compared to Sham Controls

**Figure 2:**
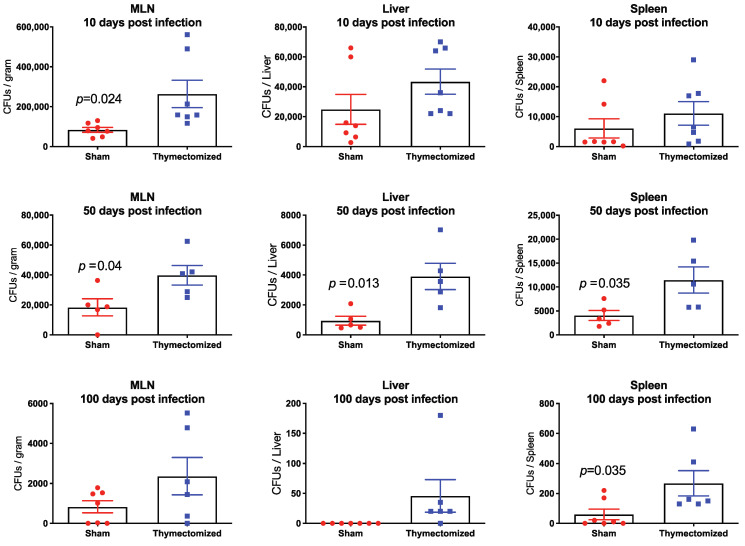
Thymectomy resulted in increased bacterial burdens in the mesenteric lymph nodes, spleens and liver during persistent *Salmonella* infection. Adult F1 mice were subjected to either full thymectomy or sham surgical control treatment. Three weeks later, mice were orally infected with WT *Salmonella* (5 × 10^7^ CFU) and bacterial burdens were assessed at 10, 50 and 100 days post infection. For bacterial burdens in the spleen and liver, the data was calculated as the number of colony forming units per organ; for the MLNs, the data was calculated as the number of colony forming units per gram. *p* values were generated using an unpaired Student’s T test and are shown for each organ where significant. Graphs are representative of two independent experiments combined and show the mean ± SEM.

We next posited that the observed increase in infection-associated mortality may be related to uncontrolled bacterial replication in the absence of thymic output. To test this, we measured bacterial burdens in the spleens, livers, and mesenteric lymph nodes (MLNs) of thymectomized and sham surgical control mice. We sought to cover the span of persistent infection and so we chose an early time after infection (10 days), a time when the persistent state is clearly established (50 days) and a very late time of persistence (100 days). When mice were thymectomized prior to infection, bacterial burdens were significantly increased in the MLNs of thymectomized mice compared to sham controls at all three time points post infection, in the spleens of thymectomized mice at 50 and 100 days post infection, and in livers at 50 days post infection (Figure 2). It was also noteworthy that by 100 days post infection, many of the sham surgical control mice exhibited bacterial burdens below the limit of detection for this assay, indicating that they were either controlling or perhaps even clearing *Salmonella* infection, while thymectomized mice demonstrated detectable bacterial burdens in all organs assessed. Reflecting what we observed with mortality (Figure 1), we did not observe any differences in bacterial burdens between thymectomized and sham surgical mice when we performed thymectomy 30 days after *Salmonella* infection (data not shown), further indicating that thymic function is protective during the initiation of *Salmonella* infection, but appears to be unnecessary once *Salmonella* infection has already proceeded into the persistent phase. 

### 2.3. Preinfection Thymectomy Induces Increased Salmonella-Specific CD4 T Cell Numbers

The increased bacterial burdens and subsequent mortality led us to speculate that the CD4 T cell response was blunted in thymectomized mice, which prevented control of the infection. To determine this possibility, we first had to define the *Salmonella*-specific CD4 T cell response in thymectomized and sham surgical control mice throughout the time course of persistent infection. To achieve this, we made use of a *Salmonella* strain that was tagged genetically at the C-terminus of the *Salmonella* outer membrane porin C (OmpC) with a well-known CD4^+^ T cell epitope called 2W1S (EAWGALANWAVDSA—henceforth, OmpC-2W1S). We and others have used this strain to analyze endogenous anti-OmpC-2W1S CD4^+^ T cell responses by employing MHC class II tetramers expressing the 2W1S peptide that can be used to track *Salmonella*-specific CD4 T cell kinetics over the course of persistent infection [20,28,29]. To assess the T cell response to persistent infection, mice were orally infected with 5 × 10^7^ CFU of the OmpC-2W1S *Salmonella* three weeks after surgical treatment (sham or thymectomy), as previously described. Spleens and MLNs were harvested at 10, 50 and 100 days post infection and *Salmonella*-specific CD4^+^ T cell responses were quantified using the 2W1S-MHC Class II tetramer and magnetic bead enrichment prior to flow cytometry analysis as we have done previously [21,22]. 

**Figure 3:**
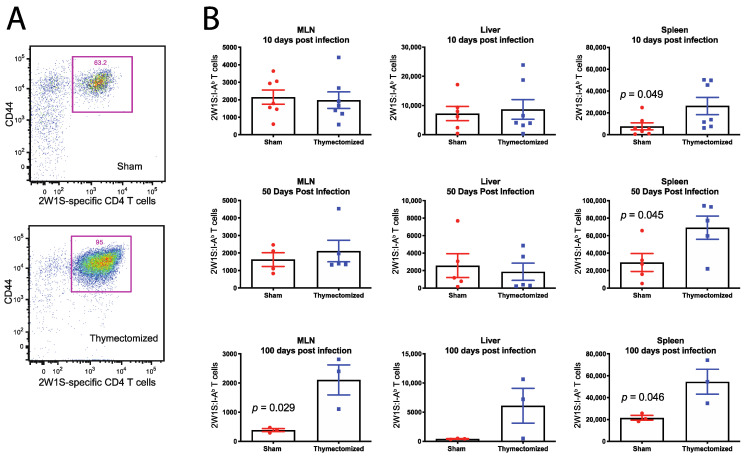
Thymectomy prior to infection results in increased numbers of *Salmonella*-specific CD4^+^ T cells during persistent *Salmonella* infection. Adult F1 mice were subjected to either full thymectomy or sham surgical control treatment. Three weeks later, mice were orally infected with 5 × 10^7^ CFU of OmpC-2W1S *Salmonella*. At 10, 50 and 100 days post infection, 2W1S-specifc CD4^+^ T cells were enriched and quantified in the mesenteric lymph nodes (MLNs), spleens, and livers using 2W1S:I-A^b^ tetramers. (**A**) Representative flow plot of antigen-specific CD4^+^ T cells in the spleen, 50 days post infection is shown for sham and thymectomized groups. (**B**) The total number of 2W1S-specific CD4^+^ T cells at 10, 50 and 100 days post infection are shown for each organ. p values were generated using unpaired Student’s T test and are shown where significant for each group. Graphs are representative of 3–4 independent experiments and show the mean ± SEM.

Surprisingly, when we assessed the *Salmonella*-specific CD4^+^ T cell response in thymectomized and sham surgical control mice, we observed thymectomized mice exhibited significantly higher numbers of *Salmonella*-specific CD4^+^ T cells in the spleen at 10, 50 and 100 days post infection, and in the MLN at 100 days post infection (Figure 3). As before, when we performed thymectomy 30 days after *Salmonella* infection, we did not observe any differences in the numbers of *Salmonella*-specific CD4^+^ T cells between thymectomized or sham surgical control mice (data not shown), indicating that this expansion happens early after infection in thymectomized mice.

**Figure 4:**
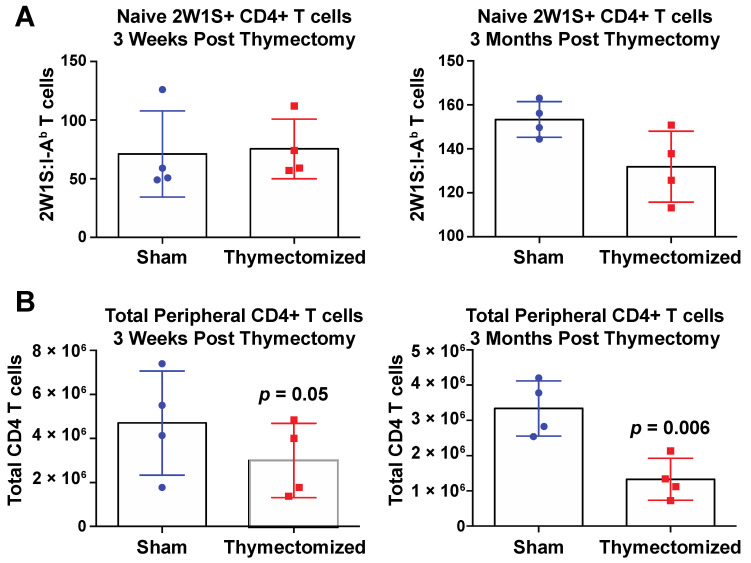
Thymectomy in the absence of infection does not lead to 2W1S^+^ CD4^+^ T cell expansion but does decrease total CD4 T cell numbers. Adult F1 mice were subjected to either full thymectomy or sham surgical control treatment. The (**A**) 2W1S^+^ and (**B**) total CD4^+^ T cells were measured in all peripheral lymph nodes and spleen three weeks or three months post surgery using 2W1S:I-A^b^ tetramer and magnetic activated cell sorting techniques (Miltenyi). The 2W1S-specific CD4^+^ T cells were enumerated via flow cytometry and p values were generated using unpaired T test and are shown. Representative of 2–3 independent experiments for each time point.

The elevated level of *Salmonella*-specific CD4^+^ T cells we measured in thymectomized mice was counterintuitive, since thymectomized mice are deficient in overall peripheral CD4^+^ T cells [30,31]. To ascertain whether there was a difference between thymectomized or sham control mice at baseline, we measured the number of both 2W1S-specific and total CD4^+^ T cell numbers in thymectomized and sham surgical control mice in the absence of *Salmonella* infection. This would allow us to discern whether or not the observed increase in *Salmonella*-specific CD4^+^ T cells measured in thymectomized mice was a function of thymectomy alone, or a response to infection. We found that thymectomized mice had slightly lower, but not significantly different, numbers of peripheral, naive 2W1S-specific CD4^+^ T cells compared to sham surgical controls at both three weeks and three months post thymectomy (Figure 4A). We also observed thymectomized mice had significantly lower overall numbers of peripheral CD4^+^ T cells compared to sham surgical controls as expected (Figure 4B). This shows that the increased *Salmonella*-specific CD4^+^ T cells were not due to a baseline increase in naïve precursor T cells in response to thymectomy but was due to an increased response to infection itself.

### 2.4. Peripheral Salmonella-Specific CD4 T Cells in Thymectomized Mice Are Deficient in Production of IL-17

The observation that thymectomized animals have similar numbers of 2W1S-specific CD4^+^ T cells as sham surgical control mice at the time point coinciding with the start of *Salmonella* infection indicates that the elevated 2W1S-specific CD4^+^ T cells in thymectomized animals is in response to *Salmonella* infection, not thymectomy alone. Elevated *Salmonella*-specific CD4^+^ T cells in thymectomized mice are most likely a response to increased antigenic load from increased bacterial burdens, creating a feedback loop that then leads to greater expansion of *Salmonella*-specific CD4^+^ T cells throughout persistent time points of infection. We hypothesized that these T cells were somehow impaired in their effector function.

**Figure 5:**
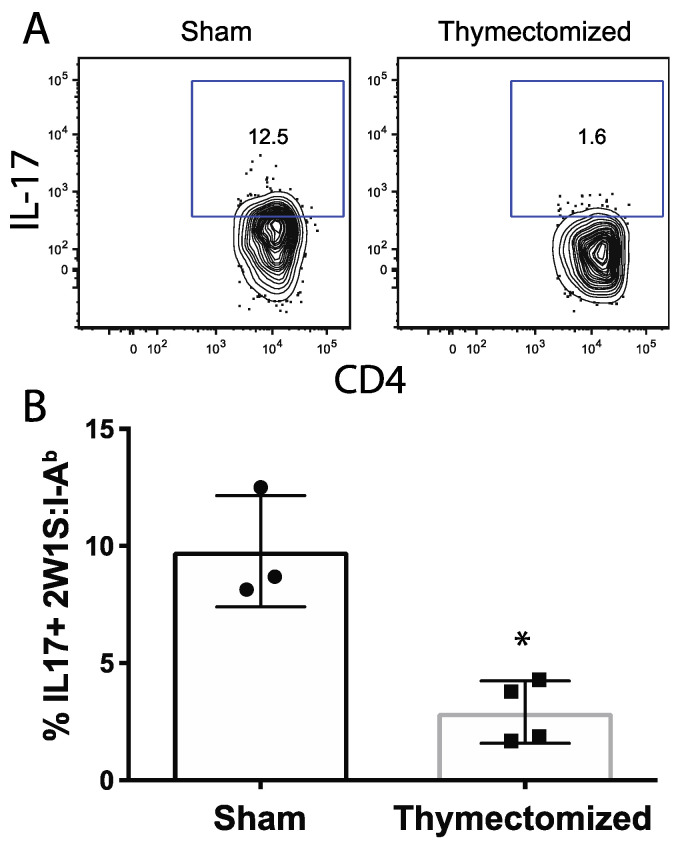
Thymectomy prior to *Salmonella* infection reduces IL-17 in the *Salmonella*-specific CD4 T cells. Adult F1 mice were subjected to either full thymectomy or sham surgical control treatment. Three weeks later, mice were orally infected with 5 × 10^7^ CFU of OmpC-2W1S *Salmonella*. Ten days post infection, in vivo peptide restimulations were performed to assess cytokine production. (**A**) Representative flow plot of 2W1S-specific CD4^+^ T cells in the MLN showing IL-17 expression. Cells shown are gated initially on lymphocytes and then on CD4^+^ lineage^-^ cells and finally, of the CD4^+^ population, CD44^hi^ and 2W1S:I-A^b^ tetramer^+^. The 2W1S-specific cells are shown as IL-17-expressing, CD4^+^ cells. Cytokine gates are set on the CD44^-^ naïve population. (**B**) Quantification of IL-17-producing 2W1S-specific CD4^+^ T cells is shown. P values were generated using unpaired Student’s T test and are shown. * = *p* < 0.005. Representative of 2 independent experiments.

To test this, we performed in vivo cytokine restimulation of 2W1S-specific CD4 T cells by intravenously administering 2W1S peptide to thymectomized and sham surgical mice 10 days post OmpC-2W1S *Salmonella* infection. Mice were euthanized two hours after peptide introduction and intracellular cytokines were measured in the 2W1S-specific CD4 T cells, as previously described [32]. We analyzed the anti-microbial cytokines IFN-γ and IL-17. Only IL-17 was consistently and significantly lower in thymectomized mice compared to the sham controls 10 days after infection (Figure 5). This observation indicated that some early effector responses in thymectomized mice were affected by the loss of thymic output and that mature naïve CD4 T cells could not respond to infection as potently as when RTEs are present during the initiation of infection.

## 3. Discussion

In the current study, we sought to determine the impact of thymic output on the establishment of a persistent *Salmonella* infection by thymectomizing resistant mice and assessing the impact of the lack of a thymus on infection. We found that thymectomy led to impaired survival during persistent *Salmonella* infection that is normally well tolerated by F1 resistant mice. It was clear that this increase in mortality was somehow related to the absence of thymic function when challenged with *Salmonella* infection. The cause of lethality was likely due to the inability of the immune response to control the infection, as bacterial burdens were higher throughout the course of infection in all organs evaluated. Notably, the *Salmonella*-specific CD4^+^ T cell response was significantly increased in mice lacking a thymus compared to sham control mice, indicating that, although there were likely a sufficient number of T cells available to control infection, they were incapable of doing so. As far as we know, this is the first study to directly measure the outcome of thymectomy on enteric bacterial infection and the role for the T cell response in a bacterial infection thymectomy model.

Importantly, thymic output appeared to be essential only when infection was initiated but was dispensable once infection was already established, indicating that the thymus likely aided in controlling the early establishment of infection. There are several potential explanations for why this may have occurred. It is known that thymic function is disrupted during times of immunological stress and infection [9,33,34]. It is possible that ongoing persistent *Salmonella* infection resulted in decreased thymic function and infection-related thymic involution. Thus, when the thymus was removed during surgery, the contribution of the thymus in protective immunity was already suppressed to a degree that its removal had a negligible impact on disease outcomes. Conceptually, that *Salmonella* infection resulted in decreased thymic function prior to thymectomy contradicts previously published data suggesting that *Salmonella* infection has no observable impact on thymic output despite observable thymic atrophy [12]. 

It is also possible that our findings could be explained by a unique protective role for thymic output, particularly RTEs, during early time points of infection. If this were true, then loss of thymic function prior to infection would remove a crucial population of lymphocytes necessary for early bacterial control. It is conceivable that once the infection is established and expansion of *Salmonella*-specific CD4^+^ T cells has occurred in the periphery, the immune response is established, and the contribution of RTEs is minimal, thus thymic removal has no measurable impact on infection outcomes. It is known that CD8^+^ RTEs express the markers necessary for intestinal homing and migrate directly to the small intestine epithelium upon exiting the thymus [35]. Therefore, it is plausible that CD4^+^ RTEs also migrate to the intestine and are present at the site of the initial immune encounter with *Salmonella*, perhaps playing a critical role in combatting the early infection; however, it is not currently known whether CD4^+^ RTEs migrate into intestines similarly to CD8^+^ RTEs. Removal of thymic output could eliminate this population from the small intestine, resulting in increased susceptibility to infection. In a scenario where thymic function is removed after the establishment of the infection, bacteria have already disseminated systemically and RTEs may not be as important in protection at the intestinal mucosa, thus removal of the thymus is of little consequence. 

We also observed that, while at higher total numbers, *Salmonella*-specific CD4 T cells from thymectomized mice produced significantly less IL-17 compared to sham control mice, implying that the Th17 subset in these mice was disrupted. Th17 immunity is known to enhance barrier integrity at mucosal sites and has been demonstrated to be protective against many enteric pathogens including *Salmonella* [36,37]. IL-17A-deficient mice demonstrate a modest increase in bacterial dissemination following oral *Salmonella* infection, suggesting a protective role for IL-17 in the maintenance of the mucosal barrier at early infection time points [38]. In non-human primate models, blunted intestinal Th17 responses allowed for increased bacterial burdens in the MLNs of SIV–*Salmonella*-co-infected animals [39]. Other studies have identified another population of effector cells, termed natural Th17 cells (nTh17), which are capable of producing IL-17 in the absence of antigen [40,41]. These nTH17 cells have been identified in both thymic and peripheral lymphoid tissues. Our observation that *Salmonella*-specific CD4 T cells from thymectomized animals were impaired in the ability to produce IL-17 could be reflective of the loss of nTh17 cells in these animals. Notably, IL-17 is important for neutrophil recruitment to sites of inflammation. During *Salmonella* infection, the early innate immune responses initiated in the Peyer’s Patches and MLNs involve the recruitment of neutrophils and inflammatory monocytes, which help slow the spread of bacteria to systemic tissues [42,43,44]. Indeed, it has been reported that neutrophil depletion leads to increased bacterial loads of *Salmonella* in the liver, suggesting that neutrophils play an important role in the prevention of bacterial dissemination to systemic sites [45]. The importance of neutrophils during early *Salmonella* infection is further highlighted in a study which demonstrated that neutrophils are a key source of cellular IFN-γ during the acute phase of *Salmonella* infection [46]. Our current study aligns with this paradigm, especially considering that the loss of thymic output affecting IL-17 production is important early during *Salmonella* infection but, is less important later when neutrophils are not expected to play as substantial a role in controlling infection.

One application for the current study may lie in a better understanding of immunological aging and immunosenescence and how these conditions affect infectious immunity. Decreases in overall thymus size, alterations in thymic cellular composition and significant decreases in thymic output are known to occur as people age [47,48,49]. Simultaneously, as people age, they become more prone to infection, experience decreased responsiveness to vaccination and are more prone to autoimmune disorders [50,51]. While multiple cell types, both innate and adaptive, have been implicated in immunosenescence, T cells are known to experience several significant changes in function and frequency with age. For one, the peripheral T cell compartment shows reduced TCR diversity [50,52]. Additionally, there is a markedly decreased functional capacity and production of naïve T cells, and an accumulation of non-functional or anergic memory T cells [51,52]. Many of these functional impairments can be directly tied back to thymic function, particularly the maintenance of peripheral TCR diversity and the production of naïve CD4^+^ T cells [48,53]. In our experimental model of thymectomy during infection, we observed potential decreases in cytokine production capacity by CD4^+^ T cells in thymectomized animals, which mirrors the diminished functional capacity of naïve T cells seen with aging in humans. It is possible that thymectomizing mice prior to infection effectively induced accelerated immunological aging by removing the production of an, as yet, unappreciated subset of naïve CD4^+^ T cells and caused a loss of TCR diversity, as well as impairing effector function in the naïve peripheral lymphocyte compartment. Notably, it is known that both older mice and humans are more susceptible to severe disease when infected with *Salmonella* and this may be related to the findings presented here in regard to aging contributing to the loss of thymic output [54,55,56,57,58].

In the absence of inflammation and under homeostatic conditions, RTEs are prone to anergy; however, in the face of inflammation, RTEs are capable of mounting appropriate responses [59]. CD4^+^ RTEs may be preferentially homing to intestinal mucosal sites during their three-week period of peripheral development [1,35]. These mucosal tissues are key sites of antigen presentation, both from commensal organisms, as well as potential pathogens. Perhaps these tissues are where CD4^+^ RTEs complete post thymic maturation. In the absence of infection or inflammation, they may become tolerized against self-antigens or antigens from commensal microbiota. Alternatively, in the presence of inflammation, as would be the case at the initiation of a *Salmonella* infection, RTEs become robust effector cells that are essential mediators of the early protective immune responses against this persistent pathogen. Further studies are needed to directly identify the exact role of RTEs in controlling *Salmonella* infection, particularly at the portals of entry such as the intestine.

## 4. Materials and Methods

### 4.1. Mouse Line Usage and Rationale

C57BL/6J and 129x1/SvJ mice were originally purchased from Jackson Laboratory (Bar Harbor, ME) and were bred and maintained in specific pathogen-free conditions in the vivarium at Tulane University School of Medicine under the guidance of the Department of Comparative Medicine. C57BL/6J and 129x1/SvJ were then bred to generate F1 hybrid mice. This study was approved by Animal Institutional Care and Use Committee (The IACUC protocol number is 4173R). 

### 4.2. Thymectomy Procedure

The 6-week-old, F1 mice were used for all thymectomies and sham surgical control. Mice were sedated using isoflourane, the surgical incision site was prepped, and a superficial midline incision was made to expose the sternum. The sternum was cut midline from the top of the sternum to the second rib to expose the thymus. The thymus was carefully removed with forceps in the case of fully thymectomized animals or physically agitated with forceps in the case of sham surgical controls. Immediately after removing or agitating the thymus, the superficial skin was closed using wound clips. Mice were then administered 100 μL Carprophen (5 mg/mL) at the surgical site and an additional 100 μL Buprenorphine (20 μg/mL) on the rear haunch. On the following day, post operative care was administered in the form of 100 uL Carprophen at the surgical site and 100 μL Buprenorphine on the rear haunch. Then 7–10 days later, wound clips were removed.

### 4.3. Bacterial Strains and Infection

*Salmonella enterica* subsp. *Enterica* serovar Typhimurium SL1344 was obtained from Marc Jenkins (University of Minnesota, St. Paul, MO, USA). *Salmonella* was chromosomally tagged with the exogenous 2W1S peptide (EAWGALANWAVDSA), as previously described [20,60]. Briefly, primers were designed with sequences homologous to the last portion of the OmpC gene, deleting the stop codon, to a region downstream in the genome. A single FLAG epitope tag sequence was included to enable protein identification via Western Blot. Linear PCR products were generated by amplification with *Pfx* Platinum Taq Polymerase (Invitrogen, Carlsbad, CA, USA) from plasmid DNA isolated from pJM1. PCR amplification products were purified using commercial PCR clean-up columns (Qiagen) and were subsequently used for electrotransformation. *Salmonella* containing the temperature-sensitive pKD46 plasmid, carrying bacteriophage λ red genes, were made electrocompetent by growth at 37 °C in LB medium supplemented with 100 μg/mL ampicillin and 25 mM arabinose to an OD_600_ of O.5. Cells were pelleted via centrifugation, washed three times in ice-cold 10% glycerol, and concentrated to 50 μL. Bacterial suspensions were mixed with 0.5–1 μg of PCR product and incubated on ice for 30 min before transferring to a chilled 0.2-cm cuvette. Cuvettes were subjected to a single pulse of 12.5 kV/cm. After recovering for 1 h at 37 °C in SOC medium, bacteria were plated on LB agar plates supplemented with 10 μg/mL kanamycin. Bacteria were allowed to grow for ~18 h, before 4 colonies were selected for verification of recombination. DNA sequencing, Western Blot analysis, and in vivo expansion of 2W1S-specific CD4 T cells were evaluated to verify successful recombination. OmpC-2W1S bacteria were tested in vitro for attenuation by comparing their growth curves to wild-type SL1344 *S.* Typhimurium. 

For bacterial burden studies and survival studies, mice were infected with wild-type *S.* Typhimurium SL1344 via oral gavage. Bacteria were grown to late stationary phase via overnight incubation in a 37 °C water bath in LB supplemented with Streptomycin 50 μg/mL. Bacteria were pelleted at 4000 RPMs, excess media was removed, and the bacteria were resuspended in PBS to an OD_600_ of 0.5. The effective dose of bacteria administered to mice was verified by plating bacteria on Luria broth agar and incubating overnight at 37 °C.

Before infection, food was withheld from the mice for a 16–18 h period to reduce gastric acidity and enhance infectivity. Mice were given an estimated dose of 5 × 10^7^ CFU. 

For all antigen-specific studies using the 2W1S MHC Class II epitope, mice were infected with OmpC-2W1S *S.* Typhimurium. Bacteria were grown in the same manner as described above. However, antibiotic selection was achieved using Kanamycin to select for recombinantly tagged bacteria. 

### 4.4. Bacterial Plating

In order to quantify the bacterial load in key lymphoid organs during persistent *Salmonella* infection, thymectomized or sham surgical control mice were euthanized via carbon dioxide asphyxiation. Spleen, liver and mesenteric lymph nodes were harvested, weighed, placed in PBS containing 0.1% Triton-X 100 and homogenized using a Wheaton tissue homogenizer. Homogenates were then serial diluted and plated onto LB agar plates containing 100 μg/mL streptomycin, in 100 μL volumes at 10^−2^, 10^−1^, or neat dilutions. Plates were incubated at 37 °C overnight and colonies were counted the following day. For bacterial burdens in the spleen and liver, the data was calculated as the number of colony forming units per organ; for the mesenteric lymph nodes, the data was calculated as the number of colony forming units per gram. 

### 4.5. Cell Preparations 

In order to enumerate 2W1S-specific CD4 T cells during infection, single-cell suspensions were made from the spleen, liver and mesenteric lymph nodes of infected mice. Spleens and MLNs were homogenized over a 100 μm nylon mesh filter in cold sorter buffer (1× phosphate buffered saline (PBS) containing 2% newborn calf serum (NCS) and 0.1% sodium azide (NaN_3_)). Livers were excised from mice and minced into smaller pieces using surgical scissors. Liver pieces were then incubated in RPMI media containing 1 mg/mL collagenase IV (Sigma-Aldrich, St. Louis, MO, USA) for 1 h at 37 °C and subsequently homogenized over a 100 μm nylon filter. Removal of hepatocytes from cell suspension was achieved by centrifugation at 50× *g* for 2 min. Supernatants were collected, transferred to a new 15 mL conical tube and the pelleted hepatocytes were discarded. Supernatants were centrifuged at 500× *g* to pellet the remaining cells; supernatants were discarded, and the remaining cell pellet was resuspended in Hank’s Buffered Salt Solution (HBSS). Cell suspensions were then underlayered with a 15% OptiPrep gradient (Sigma-Aldrich) prepared with HBSS and spun at 750× *g* for 20 min at room temperature, without a break. Lymphocytes were isolated from the interface of the HBSS and OptiPrep using a 1 mL pipette. Lymphocytes were transferred to a new tube and washed with 10 mL HBSS to remove any residual OptiPrep. 

### 4.6. Tetramer Labeling and Magnetic Activation Cell Sorting 

Cell suspensions prepared as described above were washed with 10 mL cold sorter buffer and centrifuged at 500× *g* to pellet cells. Samples were resuspended in 200 μL FC block (Clone 2.4G2 SFM supernatant + 2% mouse serum, 2% rat serum, 0.1% NaN_3_). Cells were then enriched for 2W1S-specificity using a protocol previously described by Moon et al. in 2007 (Moon et al., 2007). To this end, 2W1S:I-A^b^-APC MHC-II tetramer ((EAWGALANWAVDSA) was added at 10 μM per sample and incubated for 1 h at room temperature in a dark environment. After incubation, cell suspensions were washed with 15 mL ice-cold sorter buffer and spun at 500× *g* for 5 min at 4 °C. Cell pellets were then resuspended in 200 μL sorter buffer before adding 25 μL anti-APC microbeads (Miltenyi, Auburn, CA, USA) and incubating on ice in a dark environment for 30 min. After incubation, cells were rinsed with 15 mL sorter buffer and centrifuged at 500× *g* for 5 min at 4 °C. During centrifugation, a Miltenyi LS column was set up on a quadroMACS magnet (Miltenyi) and prerinsed with 3 mL sorter buffer, discarding the flow through. A new 15 mL conical tube was placed under the LS column in order to collect sample flow through. After centrifugation, supernatants were discarded and remaining cell suspension was vortexed, resuspended in 3 mL ice-cold sorter buffer and filtered through nylon mesh prior to adding cell samples to the rinsed LS column. After the 3 mL cell suspension volume was allowed to pass through the LS column completely, it was rinsed three times by applying a 3 mL volume of ice-cold sorter buffer and allowing the volume to completely pass through the column, collecting the unbound flow through in a 15 mL conical tube. To collect the bound fraction, the LS column was removed from the magnet and placed over a new 15 mL conical tube. The 5 mL sorter buffer was applied to the LS column and was forced through the column in one continuous motion using the supplied plunger. The bound and flow through samples were spun down at 500× *g* for 5 min at 4 °C.

### 4.7. Flow Cytometry

In order to enumerate and characterize the expression of cell surface markers, cell suspensions acquired in the above-described preparations were stained with fluorescently conjugated flow cytometry antibodies. To this end, cell preparations were suspended in 100 μL sorter buffer and were minimally stained with lineage-negative antibodies (CD11b, CD11c, F4-80, and CD19), CD4, and CD44. Samples were additionally stained with CD3ε, CD8α and CD69. Samples were stained for 30 min, on ice in a dark environment by applying 1 μL per antibody per sample. Cells were then rinsed in 10 mL cold sorter buffer, pelleted at 500× *g* for 5 min at 37 °C and resuspended in sorter buffer prior to FACS analysis. 

For experiments where absolute cell numbers were enumerated, 5 μL of unstained cell sample was removed, noting the total volume of the cell suspension, and added to 200 μL of AccuCheck counting beads (Invitrogen) at a known concentration. To determine the absolute number of positive cells for any given marker, the following formula was used:

$$\left(\begin{array}{c} total\;number\;of\;\\ cells\;in\;sample \end{array}\right)=\left(\frac{cell\;count}{bead\;count}\right)\left(\begin{array}{c} bead\;stock\;\\ concentration \end{array}\right)\left(\frac{bead\;volume}{cell\;volume}\right)\left(\begin{array}{c} total\;\\ sample\;volume \end{array}\right)$$

Cells were collected on a Fortessa (Beckton-Dickson, Franklin Lakes, NJ, USA) equipped with four lasers (405, 488, 561 and 640 nm). Data was analyzed using FlowJo software (TreeStar, Ashland, OR, USA). Tetramer-positive cells were gated as “Dump” negative (CD11b^−^, CD11c^−^, F4-80^−^, CD19^−^), CD3ε^+^, CD8α^−^, CD4^+^, CD44^hi^ and 2W1S:I-A^b^-APC^+^. 

### 4.8. In Vivo Cytokine Stimulations

In order to study cytokine production in vivo, 100 μg 2W1S (EAWGALANWAVDSA) peptide was administered IV via retro-orbital injection to *Salmonella*-infected mice and allowed to circulate for 2 h. After stimulation, mice were sacrificed via carbon dioxide asphyxiation and spleen and MLNs were processed and prepared for flow cytometry analysis, as previously described. 

### 4.9. Intracellular Cytokine Staining

After 2 h of peptide stimulation in vivo, animals were sacrificed, and lymphoid organs were harvested and immediately placed into complete RPMI media containing 10 μg/mL Brefeldin A (Sigma-Aldrich) and subsequently prepared into a single-cell suspension, as previously described. During all steps of cell preparation, surface staining and tetramer labeling cells were maintained in 10 μg/mL Brefeldin A to prevent cytokine export. For surface staining procedures, cells were blocked with FcBlock and stained with anti-mouse surface antibodies, as previously described. After surface staining and tetramer labeling, cells were fixed and permeabilized using the BD Cytofix/Cytoperm kit, according to the manufacturer’s instructions, and stained overnight for intracellular cytokines. All antibodies were purchased from either Biolegend or eBioscience. On the following day, samples were washed twice with 1× permeabilization wash buffer and resuspended in sorter buffer for FACS analysis. 

### 4.10. Data Analysis

All data was analyzed using GraphPad Prism. For survival analysis, statistical significance between surgical groups was determined using the Gehan–Brelow–Wilcoxon test. For bacterial burden comparisons and 2W1S-specific CD4^+^ T cell comparisons, statistical significance between surgical groups was determined using an unpaired Student’s T test. For cytokine comparisons and transcription factor expression, statistical significance between surgical groups was determined using an unpaired Student’s T test.
